# Intra-arterial cocktail therapy for patients with anterior circulation large vessel occlusion who achieved endovascular reperfusion

**DOI:** 10.3389/fneur.2024.1450156

**Published:** 2024-12-06

**Authors:** Zi-Ai Zhao, Hai-Zhou Hu, Wei Li, Jing Qiu, Yong-Gang Zhao, Thanh N. Nguyen, Hui-Sheng Chen

**Affiliations:** ^1^Department of Neurology, General Hospital of Northern Theater Command, Shenyang, China; ^2^Department of Neurology and Radiology, Boston Medical Center, Boston, MA, United States

**Keywords:** clinically ineffective reperfusion, endovascular treatment, large vessel occlusion, cerebroprotection, cocktail therapy

## Abstract

**Background:**

Clinically ineffective reperfusion (CIR) refers to the discrepancy between successful reperfusion and a favorable functional outcome in patients with large vessel occlusion (LVO) stroke after endovascular treatment (EVT). The Improving Neuroprotective Strategy for Ischemic Stroke with Sufficient Recanalization after Thrombectomy by Intra-arterial Cocktail Therapy (INSIST-CT) trial aimed to explore the safety, feasibility, and efficacy of intra-arterial cocktail therapy using argatroban, dexamethasone, and edaravone in patients who achieved sufficient reperfusion after EVT.

**Methods:**

In this prospective, single-arm, pilot study, eligible patients with anterior circulation LVO who achieved sufficient reperfusion after EVT were enrolled in the INSIST-CT trial. Consecutive patients who met the inclusion/exclusion criteria were included in the control group retrospectively. In the INSIST-CT group, argatroban, dexamethasone, and edaravone were continuously administered for 30 min into the culprit artery after sufficient recanalization. The primary endpoint was the proportion of favorable functional outcome, defined as a modified Rankin Scale (mRS) score of 0–2 at 90 days. The primary safety outcome was symptomatic intracranial hemorrhage (sICH). Propensity score matching (PSM) and inverse probability of treatment weighting (IPTW) analyses were performed to account for multiple confounders.

**Results:**

A total of 30 patients were included in the INSIST-CT group, and 261 patients were included in the control group. The proportion of the patients with the primary endpoint was 60% in the INSIST-CT group and 55.9% in the control group (unadjusted odds ratio [OR] 1.18, 95% CI 0.55–2.61, *p* = 0.67; adjusted OR 1.42, 95% CI 0.62–3.26, *p* = 0.41). No significant difference in sICH at 48 h after treatment was observed between the two groups (unadjusted OR 0.96, 95% CI 0.15–3.56, *p* = 0.96; adjusted OR 0.82 95% CI 0.17–3.97, *p* = 0.809). Similar results were observed after the PSM and IPTW analyses.

**Conclusion:**

In anterior circulation, LVO patients who achieved sufficient reperfusion after EVT, bridging intra-arterial cocktail therapy with argatroban, dexamethasone, and edaravone may be safe and feasible. However, it did not improve the 90-day functional outcomes. A numerically higher probability of a favorable outcome in the INSIST-CT group suggests the potential promise of this cocktail therapy in reducing clinically ineffective reperfusion.

**Clinical trial registration:**

ClinicalTrials.gov, NCT04202549.

## Introduction

Endovascular treatment (EVT) is the most effective treatment for acute ischemic stroke (AIS) patients with large vessel occlusion (LVO). Although the proportion of LVO patients who achieve sufficient recanalization after EVT is more than 80%, nearly half of them remain disabled. This is defined as clinically ineffective reperfusion (CIR) (1). The mechanisms underlying CIR include the evolution of the infarction by the time of angiographic reperfusion, vessel re-occlusion, hemorrhagic transformation after EVT, and the no re-flow phenomenon in the cerebral microcirculation (1). Among these mechanisms, the no re-flow phenomenon may be a major contributor to CIR and is estimated to occur in one-third of ischemic stroke patients who undergo reperfusion (2). Previous studies have shown that multiple mechanisms contribute to the no-reflow phenomenon, including endothelial swelling, pericyte constriction, microthrombus formation, neuroinflammation, oxidative stress, and disruption of the blood–brain barrier (3, 4).

Argatroban, a selective thrombin inhibitor, directly inhibits both free and clot-associated thrombin, along with thrombin-induced events, and is widely used to treat AIS, particularly in Asian countries such as China and Japan (5–7). Several studies have demonstrated the neuroprotective effect of glucocorticoids on ischemic brain damage through anti-inflammatory mechanisms (8). Edaravone has been recommended for AIS therapy due to its ability to scavenge free radicals and prevent neuronal and endothelial cell injury caused by excessive Ca2+ influx and reactive oxygen species (9–11).

Given the differing mechanisms of action of argatroban, dexamethasone, and edaravone, their combination treatment may be more effective in improving no-reflow than any of these drugs used alone. Importantly, these drugs have been used to treat acute ischemic stroke in previous trials before the advent of EVT, while in preclinical studies, they have been shown to exert neuroprotective effects, typically using an ischemia/reperfusion injury model (12–14). Furthermore, we contended that intra-arterial administration of these drugs may offer benefits in improving no-reflow, thereby leading to an improvement in CIR. In this context, the Improving Neuroprotective Strategy for Ischemic Stroke with Sufficient Recanalization after Thrombectomy by Intra-arterial Cocktail Therapy (INSIST-CT) trial aimed to explore the safety, feasibility, and possible efficacy of intra-arterial cocktail therapy using this combination of agents after sufficient recanalization in AIS-LVO patients who received EVT.

## Methods

### Study design and participants

**Figure 1:**
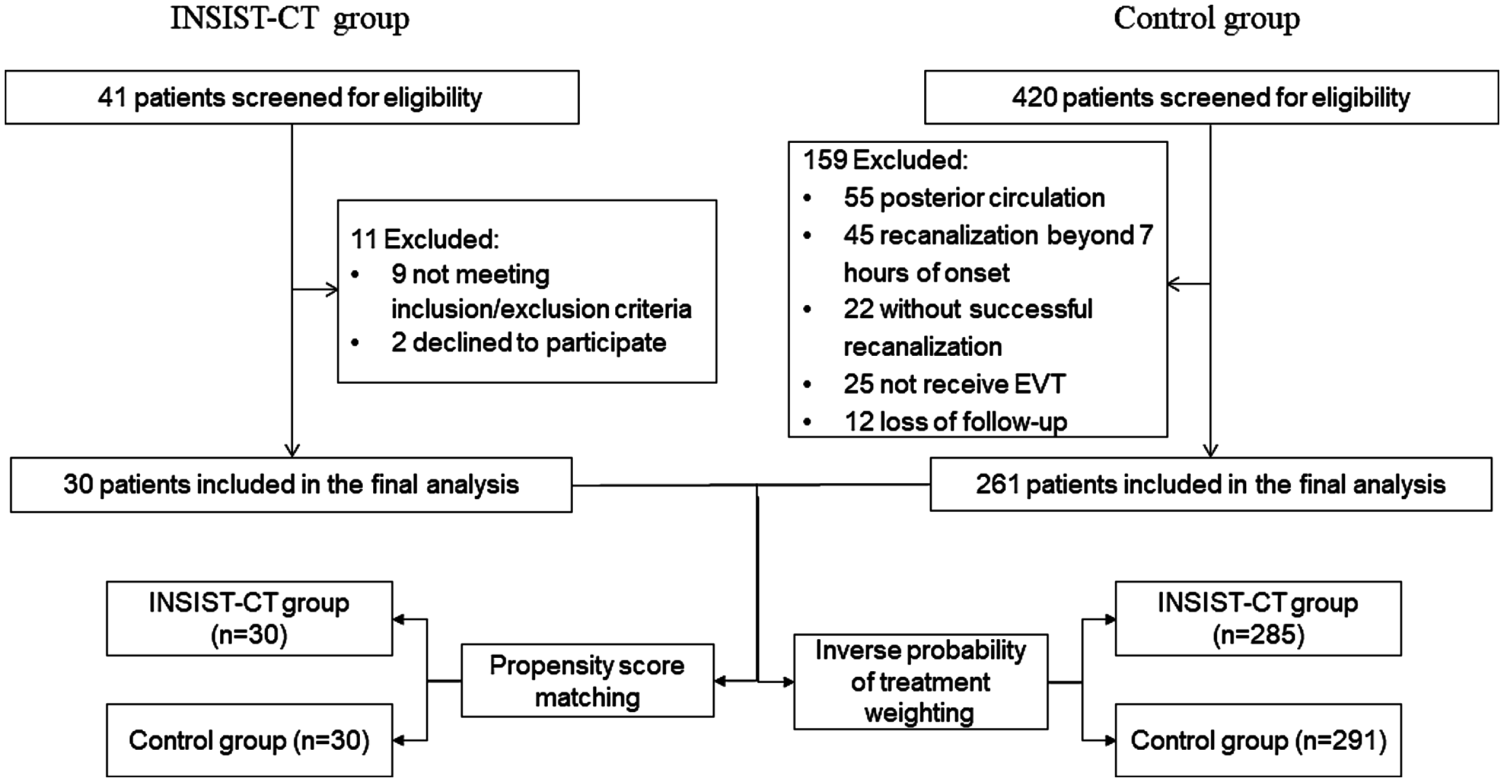
Screening and follow-up of patients.

The INSIST-CT trial was a prospective, single-arm, pilot study conducted in China from May 2020 to June 2022. It aimed to determine the safety, feasibility, and possible efficacy of intra-arterial cocktail compounds containing argatroban, dexamethasone, and edaravone, in anterior circulation AIS-LVO patients who underwent EVT. The study protocol is available in Appendix 1. During the same period, consecutive patients with anterior circulation AIS-LVO who underwent EVT were retrospectively screened as controls. Patients with a pre-stroke modified Rankin Scale (mRS) score of 0–2 and occlusion in the internal carotid artery or proximal middle cerebral artery (M1 or M2 segment) were included. The trial flowchart is shown in Figure 1. Eligible participants in the study were those with anterior circulation AIS-LVO who underwent EVT according to the AHA/ASA guidelines (15) and achieved sufficient reperfusion [modified Treatment in Cerebral Ischemia (mTICI) 2b-3] within 7 h of stroke onset. The key exclusion criteria were as follows: hemorrhagic transformation (PH2) detected on non-contrast computed tomography (NCCT) performed immediately after the procedure, severe hepatic or renal dysfunction, and severe uncontrolled hypertension (systolic blood pressure over 200 mmHg or diastolic blood pressure over 110 mmHg). The detailed inclusion and exclusion criteria are listed in Appendix 1. The INSIST-CT trial was registered on clinicaltrials.gov (NCT04202549). The protocol and data collection for the trial were approved by the ethics committee of the General Hospital of the Northern Theater Command. All patient representatives provided written informed consent before being included in the trial.

### Procedure

Once successful angiographic reperfusion (mTICI 2b-3) was achieved, intra-arterial administration of a mixture containing argatroban (0.3 mg/min), dexamethasone (0.1 mg/min), and edaravone (0.3 mg/min) was performed continuously for 30 min through a supporting catheter in the culprit artery. All patients received standard perioperative treatment according to current guidelines (15).

### Outcomes

The primary outcome was a favorable functional outcome, defined as a modified Rankin Scale (mRS) score of 0–2 at 90 days. The secondary outcomes included an excellent functional outcome (mRS 0–1) at 90 days and early neurological improvement (ENI), defined as a decrease of more than 4 points in the NIHSS score at 48 h after the treatment. The primary safety outcome was symptomatic intracranial hemorrhage (sICH), defined as an increase of ≥4 points in the NIHSS score as a result of intracranial hemorrhage, according to the ECASS-3 study (16).

As a *post hoc* analysis, the 90-day mRS distribution and the changes in the NIHSS score at 48 h and 10 days or upon discharge were examined as secondary outcomes; any instance of ICH within 48 h and mortality within 90 days were evaluated as safety outcomes.

### Follow-up procedure

Study visits were made at 48 ± 12 h, 10 ± 2 days, and 90 ± 7 days after randomization. Data regarding patient demographic characteristics, baseline NIHSS, routine laboratory tests, and neuroimaging were collected. The last known normal-to-puncture time, door-to-puncture time, puncture-to-reperfusion time, onset-to-reperfusion time, location of arterial occlusion on angiography, retrieval and recanalization techniques, number of passes, reperfusion status, and neuroimaging findings were recorded. To reduce bias, the follow-up NIHSS scores were determined by the same neurologist. The 90-day clinical assessments, including the mRS, were evaluated by a qualified individual blinded to the treatment allocation, according to a standardized procedure manual. The primary endpoint was evaluated in person or by telephone interview if an in-person evaluation was not possible. Concomitant medications and any adverse events within 90 days after randomization were recorded in detail by the investigators and adjudicated by certified assessors. In addition, continuous ECG monitoring was performed on all patients who received EVT in our intensive care unit. After transfer from the intensive care unit, 24-h Holter monitoring was routinely performed to rule out paroxysmal silent AF.

### Statistical analysis

The data were presented as mean ± standard deviation, median (IQR), or number (percentage,) as appropriate. Student’s *t*-test or Mann–Whitney U test were performed to compare continuous variables based on their normality, and Pearson’s χ^2^ test was performed for categorical variables. The primary and secondary outcomes, such as excellent functional outcome, early neurological improvement, and sICH within 48 h, were estimated using binary logistic regression adjusted for age, sex, diabetes mellitus, atrial fibrillation (AF), pre-stroke mRS, pre-treatment with IVT, and onset-to-reperfusion time (ORT).

In *post hoc* analysis, we used ordinal logistic regression to evaluate the 90-day mRS distribution, a general linear model for the changes in the NIHSS score at 48 h and 10 days or upon discharge, binary logistic regression for any ICH within 48 h, and the Cox regression model for mortality within 90 days.

As for sensitivity analyses, we performed propensity score matching (PSM) and inverse probability of treatment weighting (IPTW) analyses, accounting for multiple confounders (17, 18). The propensity score (PS) was calculated using a logistic regression model adjusted for age, baseline NIHSS, pre-treatment with IVT, mechanical thrombectomy (MT) passes, and occlusion location. This analysis was performed with 1:1 PSM based on nearest-neighbor matching with a caliper width of 0.2 times the standard deviation of the PS. We estimated the IPTW based on the PS to create a synthetic sample with baseline characteristics independent of treatment assignment and further conducted the IPTW analysis.

All statistical analyses were performed using SPSS software (version 25.0) and R software (version 4.3.2), and a two-sided test with *p* < 0.05 was considered statistically significant.

## Results

### Trial population

Between May 2020 and June 2022, 30 eligible patients with AIS-LVO who met the inclusion criteria were enrolled in the INSIST-CT trial after excluding 11 patients (Figure 1). During the same period, 420 AIS-LVO patients who received EVT were consecutively screened, and 261 patients who met the inclusion/exclusion criteria were enrolled as controls (Figure 1). The majority of the baseline demographic, clinical, imaging, and interventional characteristics were compared between the INSIST-CT and control groups, except for median age [69 (58–77) vs. 64 (56–71), *p* = 0.032], sex (male, 56.7% vs. 78.9%, *p* = 0.008), and history of atrial fibrillation (63.3% vs. 41.0%, *p* = 0.019) (Table 1). The median time from the onset to reperfusion was 339 (240–525) min in the INSIST-CT group compared to 430 (310–569) min in the control group (*p = 0.073*). A total of four patients (13.3%) in the INSIST-CT group and 80 patients (30.7%) in the control group received intravenous alteplase before EVT. All patients were treated with EVT under local anesthesia. The baseline characteristics after propensity score matching are shown in Supplementary Table S1 (Appendix 2). The baseline characteristics were well balanced after the inverse probability of treatment weighting adjusted for age, pre-treatment with IVT, baseline NIHSS, MT passes, and occlusion location (Supplementary Table S2; Appendix 2).

**Table 1:** Baseline characteristics.

Variables	Total (*n* = 291)	INSIST-CT group (*n* = 30)	Control group (*n* = 261)	*p* value
Age, years	65 (56–72)	69 (58–77)	64 (56–71)	0.032
Male patients	222 (76.3)	17 (56.7)	205 (78.9)	0.008
Medical history				
Hypertension	155 (53.3)	20 (66.7)	135 (51.7)	0.120
Diabetes mellitus	78 (26.8)	12 (40.0)	66 (25.3)	0.085
Hyperlipidemia^*^	64 (22.5)	5 (16.7)	59 (23.2)	0.416
Coronary heart disease	56 (19.2)	8 (26.7)	48 (18.4)	0.276
Atrial fibrillation^†^	126 (43.3)	19 (63.3)	107 (41.0)	0.019
History of stroke	63 (21.6)	7 (23.3)	56 (21.5)	0.813
Cigarette smoking	160 (55.0)	13 (43.3)	147 (56.3)	0.176
Alcohol use	152 (52.2)	13 (43.3)	139 (53.3)	0.303
Pre-stroke mRS				0.074
0	267 (91.8)	25 (83.3)	242 (92.7)	
1	16 (5.5)	3 (10.0)	13 (5.0)	
2	8 (2.7)	2 (6.7)	6 (2.3)	
Baseline NIHSS	15 (12–18)	16 (12–19)	14 (12–17)	0.176
Baseline ASPECTS^‡^	9 (8–10)	9 (8–10)	9 (8–10)	0.387
Pre-treatment with IVT	84 (28.9)	4 (13.3)	80 (30.7)	0.077
Duration, min				
OPT	355 (240–500)	287 (183–483)	360 (243–503)	0.132
PRT	51 (40–68)	50 (43–59)	52 (39–81)	0.213
ORT	420 (305–565)	339 (240–525)	430 (310–569)	0.073
EVT				
Stent retriever	286 (98.3)	30 (100.0)	256 (98.1)	0.982
Aspiration	30 (10.3)	3 (10.0)	27 (10.3)	>0.999
Balloon angioplasty	72 (24.7)	6 (20.0)	66 (25.3)	0.525
Stenting angioplasty	21 (7.2)	1 (3.3)	20 (7.7)	0.620
MT passes, *n*	1 (1–2)	1 (1–2)	1 (1–2)	0.546
Cause of vessel occlusion				0.136
Atherosclerosis	114 (39.2)	7 (23.3)	107 (41.0)	
Cardioembolism	121 (41.6)	17 (56.7)	104 (39.8)	
Other or unknown etiology	56 (19.2)	6 (20.0)	50 (19.2)	
Occlusion location				0.978
MCA-M1	214 (73.5)	22 (73.3)	192 (73.6)	
ICA	77 (26.5)	8 (26.7)	69 (26.4)	

Data were expressed as *n* (%) or median (IQR). NIHSS, National Institute of Health Stroke Scale; ASPECTS, Alberta Stroke Program Early CT Score; IVT, Intravenous thrombolysis; OPT, Onset to puncture time; PRT, Puncture to recanalization time; ORT, Onset to recanalization time; EVT, Endovascular treatment; MT, Mechanical thrombectomy; MCA, Middle cerebral artery; ICA, Internal carotid artery.

* Seven missing data.

† Two patients with AF were classified as having an undetermined cause due to the presence of large-artery atherosclerosis.

‡ 17 missing data.

### Primary outcome

**Figure 2:**
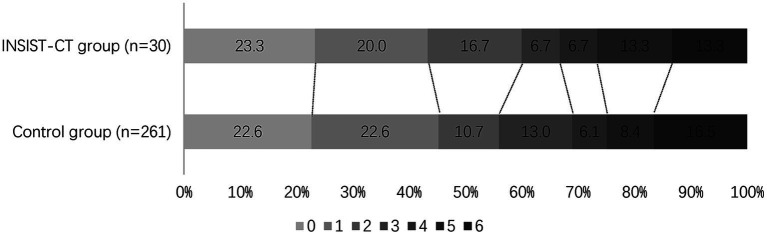
Distribution of the modified Rankin Scale (mRS) at 90 days.

**Figure 3:**
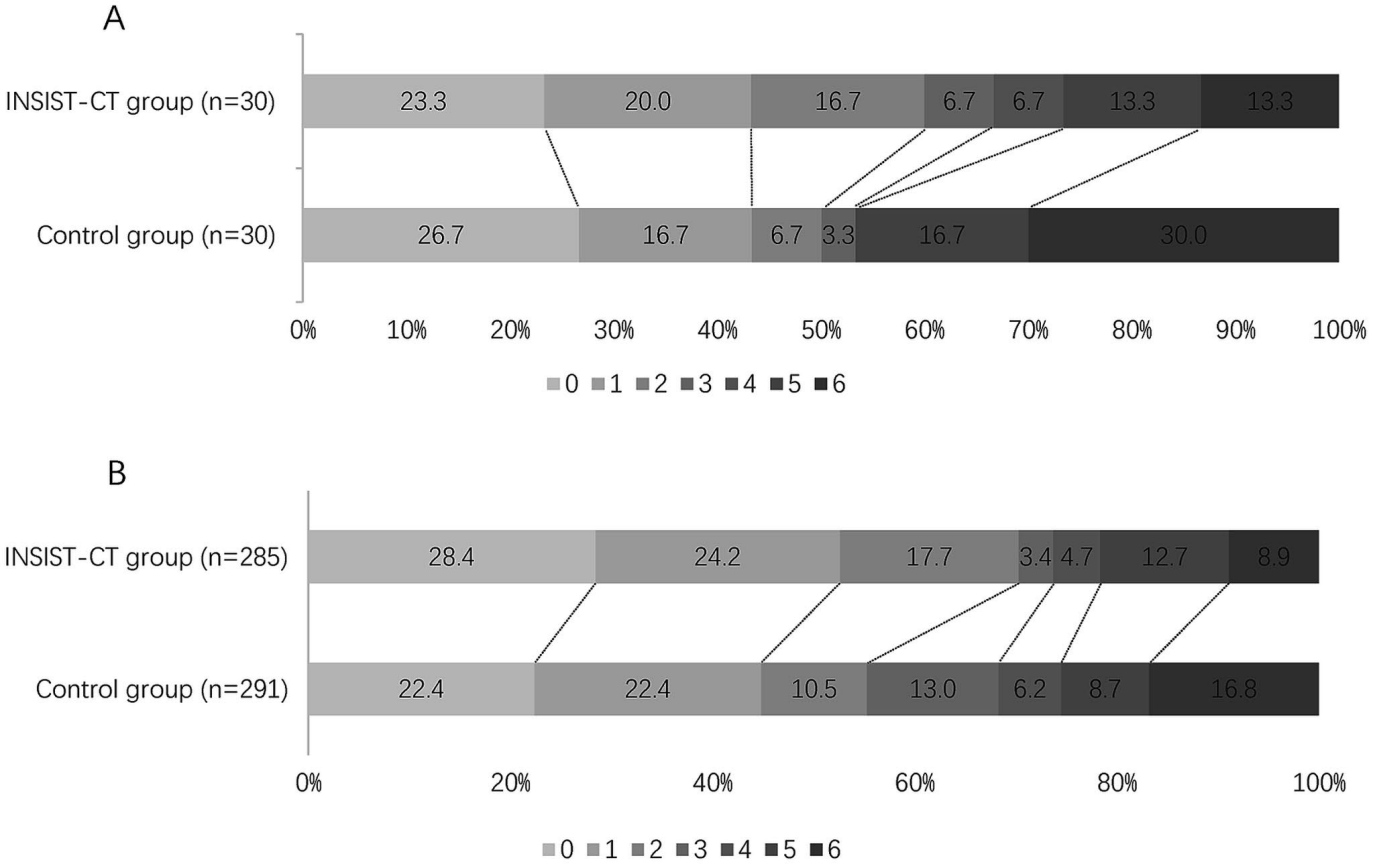
Distribution of the modified Rankin Scale (mRS) at 90 days after propensity score matching **(A)** and inverse probability of treatment weighting **(B)**.

For the primary outcome, the proportion of the patients with an mRS score of 0–2 at 90 days was 60% (18/30) in the INSIST-CT group vs. 55.9% (146/261) in the control group [unadjusted odds ratio [OR] 1.18 (0.55–2.61), *p* = 0.67; adjusted OR [aOR] 1.42 (0.62–3.26), *p* = 0.411; Table 2; Figure 2]. After the PSM, the proportion of the patients with an mRS score of 0–2 at 90 days was 60.0% (18/30) in the INSIST-CT group vs. 50.0% (15/30) in the control group [OR 1.50 (0.54–4.23), *p* = 0.44; Table 3; Figure 3A]. After the IPTW, the proportion of the patients with an mRS score of 0–2 at 90 days was 70.3% (200/285) in the INSIST-CT group vs. 55.4% (161/291) in the control group [OR 1.91 (0.83–4.42), *p* = 0.13; Table 4; Figure 3B].

**Table 2:** Primary and secondary outcomes.

Outcomes	INSIST-CT group (*n* = 30)	Control group (*n* = 261)	Unadjusted model		Adjusted model ^a^	
			OR/GMR/HR (95% CI)	*p* value	OR/GMR/HR (95% CI)	*p* value
Primary outcome						
90-day mRS 0–2	18 (60.0)	146 (55.9)	1.18 (0.55–2.61)	0.67	1.42 (0.62–3.26)	0.41
Secondary outcomes						
90-day mRS 0–1	13 (43.3)	118 (45.2)	0.93 (0.43–1.99)	0.85	1.18 (0.52–2.68)	0.70
90-day mRS distribution ^b^	2.0 (1.0–4.8)	2.0 (1.0–4.0)	1.02 (0.54–1.98)	0.95	1.31 (0.66–2.60)	0.44
ENI	17 (56.7)	116 (44.4)	1.64 (0.76–3.50)	0.20	1.62 (0.73–3.59)	0.24
Change in NIHSS ^c^						
at 48 h^†^	4.5 (0.0–7.0)	3.0 (0.0–7.0)	1.00 (0.90–1.13)	0.95	1.00 (0.89–1.12)	0.98
at 10 days or discharge^‡^	6.5 (1.0–10.8)	5.0 (0.0–10.0)	1.02 (0.89–1.18)	0.74	1.02 (0.89–1.17)	0.79
Safety outcomes						
sICH within 48 h	2 (6.7)	18 (6.9)	0.96 (0.15–3.59)	0.96	0.82 (0.17–3.97)	0.81
Any ICH within 48 h	9 (30.0)	86 (33.0)	0.87 (0.37–1.93)	0.74	1.12 (0.46–2.73)	0.80
Mortality within 90 days ^d^	4 (13.3)	43 (16.5)	0.77 (0.28–2.14)	0.61	0.54 (0.18–1.58)	0.26

Data were expressed as *n* (%) or median (IQR). CI, Confidence interval; OR, Odds ratio; GMR, Geometric mean ratio; HR, Hazard ratio; mRS, Modified Rankin Scale; ENI, Early neurological improvement; NIHSS, National Institute of Health Stroke Scale; sICH, Symptomatic intracranial hemorrhage; ICH, Intracranial hemorrhage.

a Adjusted for age, sex, diabetes mellitus, atrial fibrillation, pre-stroke mRS, and pre-treatment with IVT and ORT, which was based on the baseline characteristics at a p < 0.1 level.

b Calculated using ordinal regression analysis.

c The log (NIHSS + 1) was analyzed using a generalized linear model.

d Calculated using the Cox regression model.

† 11 missing data.

‡ 19 missing data.

**Table 3:** Primary and secondary outcomes after propensity score matching.

Outcomes	INSIST-CT group (*n* = 30)	Control group (*n* = 30)	OR/GMR/HR	*p* value
			(95% CI)	
Primary outcome				
90-day mRS 0–2	18 (60.0)	15 (50.0)	1.50 (0.54–4.23)	0.44
Secondary outcomes				
90-day mRS 0–1	13 (43.3)	13 (43.3)	1.00 (0.36–2.79)	1.00
90-day mRS distribution ^a^	2.0 (1.0–4.8)	2.5 (0.2–6.0)	1.423 (0.58–3.54)	0.44
ENI	17 (56.7)	14 (46.7)	1.495 (0.54–4.20)	0.44
Change in NIHSS ^b^				
at 48 h	4.5 (0.0–7.0)	3.0 (0.0–7.0)	1.03 (0.90–1.18)	0.64
at 10 days or discharge^†^	6.5 (1.0–10.8)	4.5 (−1.2 to 9.2)	1.07 (0.89–1.28)	0.47
Safety outcomes				
sICH within 48 h	2 (6.7)	3 (10.0)	0.64 (0.08–4.17)	0.64
Any ICH within 48 h	9 (30.0)	9 (30.0)	1.00 (0.33–3.05)	1.00
Mortality within 90 days ^c^	4 (13.3)	9 (30.0)	0.40 (0.12–1.29)	0.13

Data were expressed as *n* (%) or median (IQR). CI, Confidence interval; OR, Odds ratio; GMR, Geometric mean ratio; HR, Hazard ratio; mRS, Modified Rankin Scale; ENI, Early neurological improvement; NIHSS, National Institute of Health Stroke Scale; sICH, Symptomatic intracranial hemorrhage; ICH, Intracranial hemorrhage.

Used 1:1 propensity score matching with a caliper width of 0.2, matching for age, baseline NIHSS, pre-treatment with IVT, MT passes, and occlusion location.

a Calculated using ordinal regression analysis.

b The log (NIHSS + 1) was analyzed using a generalized linear model.

c Calculated using the Cox regression model.

† Two missing data.

**Table 4:** Primary and secondary outcomes after inverse probability of treatment weighting.

Outcomes	INSIST-CT group (*n* = 285)	Control group (*n* = 291)	OR/GMR/HR	*p* value
			(95% CI)	
Primary outcome				
90-day mRS 0–2	200 (70.3)	161 (55.4)	1.91 (0.83–4.42)	0.13
Secondary outcomes				
90-day mRS 0–1	150 (52.6)	131 (44.9)	1.36 (0.58–3.23)	0.48
90-day mRS distribution ^a^	1.0 (0.0–3.4)	2.0 (1.0–5.0)	1.30 (0.98–1.73)	0.07
ENI	161 (56.4)	129 (44.4)	1.67 (0.68–3.83)	0.27
Change in NIHSS ^b^				
at 48 h	5.1 (0.0–7.0)	3.0 (0.0–7.0)	1.03 (0.93–1.14)	0.55
at 10 days or discharge	7.0 (1.0–9.7)	5.0 (0.0–10.0)	1.08 (0.95–1.24)	0.25
Safety outcomes				
sICH within 48 h	12 (4.3)	20 (6.8)	0.62 (0.13–2.93)	0.54
Any ICH within 48 h	106 (37.4)	95 (32.7)	1.23 (0.48–3.12)	0.67
Mortality within 90 days ^c^	25 (8.9)	49 (16.8)	0.48 (0.16–1.48)	0.20

Data were expressed as *n* (%) or median (IQR). CI, Confidence interval; OR, Odds ratio; GMR, Geometric mean ratio; HR, Hazard ratio; mRS, Modified Rankin Scale; ENI, Early neurological improvement; NIHSS, National Institute of Health Stroke Scale; sICH, Symptomatic intracranial hemorrhage; ICH, Intracranial hemorrhage.

Weighted by age, baseline NIHSS, pre-treatment with IVT, MT passes, and occlusion location.

a Calculated using ordinal regression analysis.

b The log (NIHSS + 1) was analyzed using a generalized linear model.

c Calculated using the Cox regression model.

### Secondary outcomes

Similar to the primary outcome, no significant differences were observed in the secondary outcomes between the two groups, including the proportion of the patients with an mRS score of 0–1 at 90 days, and early neurological improvement. As for the exploratory secondary outcomes, no significant differences between the two groups were observed, including the mRS distribution at 90 days, the proportion of patients with an mRS score of 0–3 at 90 days, and the changes in NIHSS score compared to the baseline at 48 h and 10 days or upon earlier discharge (Tables 2–4; Figures 2, 3).

### Safety outcomes

As for the safety outcomes, no significant differences were observed in both the unadjusted and adjusted analyses of sICH at 48 h after EVT between the two groups. For the exploratory safety outcomes, including any ICH within 48 h and mortality within 90 days, no significant difference was observed between the two groups (Tables 2–4).

## Discussion

This prospective, single-arm, pilot study conducted in China explored the safety, feasibility, and efficacy of sufficient EVT reperfusion followed by an intra-arterial cocktail therapy in patients with anterior circulation AIS-LVO. The results showed that intra-arterial administration of argatroban, dexamethasone, and edaravone may be safe and feasible, but it did not improve the clinical outcomes in the population of this study compared to the controls. A numerically higher probability of a favorable outcome and a numerically lower risk of SICH were observed in the INSIST-CT group compared to the control group, which was similar to the results of propensity score matching and inverse probability of treatment weighting.

Despite improvements in EVT techniques and a high rate of reperfusion, CIR remains a serious clinical problem in approximately half of AIS-LVO patients after EVT (19–21). Factors prior to EVT, including large infarct volume, poor collateral circulation, female sex, comorbidities, admission systolic blood pressure, occlusion site, and non-bridging therapy, have been reported to be associated with CIR (22–24). After EVT, no-reflow is closely associated with CIR due to the production of harmful free radicals, aggravation of inflammation, and microthrombi (1). In this trial, an intra-arterial mixture of argatroban, dexamethasone, and edaravone was administered after successful EVT reperfusion to address the mechanisms underlying no-reflow, including the anti-inflammatory effect of dexamethasone, the inhibitory effect of argatroban on microthrombi, and the free radical scavenging effect of edaravone. These agents have shown promising results in their respective clinical trials in ischemic stroke patients. The MARVEL randomized trial found that intravenous methylprednisolone as an adjunct to EVT in patients with AIS-LVO significantly reduced the occurrence of sICH within 48 h and mortality at 90 days; however, it did not reduce 90-day disability (25). In patients with AIS with early neurological deterioration, treatment with argatroban and antiplatelet therapy resulted in better functional outcomes at 90 days (26). Our ARAIS trial showed that, among patients with acute ischemic stroke, treatment with argatroban and intravenous alteplase, compared to alteplase alone, was safe but did not improve the functional outcomes at 90 days (7). The TASTE and TASTE-SL trials found that intravenous or sublingual ED administration resulted in better 90-day functional outcomes (mRS 0–1) in AIS patients who did not receive thrombolysis or EVT and were presented within 48 h of symptom onset (27, 28). In contrast to these studies, we chose an intra-arterial cocktail strategy containing argatroban, dexamethasone, and edaravone, given the potentially better effect of intra-arterial delivery and their mechanisms to target the no-reflow phenomenon. Compared to the controls, the cocktail therapy resulted in a numerically higher probability of a favorable outcome and a numerically lower risk of sICH, suggesting the potential benefit. This finding is also in agreement with the CHOICE trial, which indicated that the use of adjunct intra-arterial alteplase improved clinical outcomes among AIS-LVO patients who had successful reperfusion following thrombectomy (29). Unlike patients who achieved successful recanalization, other studies have indicated that anti-platelet drug treatments, such as arterial and intravenous tirofiban, may benefit many patients after unsuccessful mechanical thrombectomy (30–32). Collectively, the promising results further support the bridging therapeutic strategies targeting multiple mechanisms of reperfusion injury and no-reflow in AIS-LVO patients who have had sufficient reperfusion to improve clinical outcomes.

Although, to the best of our knowledge, the INSIST-CT trial is the first attempt to improve CIR with intra-arterial cocktail therapy, it has several limitations. First, this was a single-arm trial where a concurrent control arm was used, which could have introduced potential bias and confounding effects. This single-arm design also made it impossible for the control group to receive an intra-arterial injection of saline in the same amount. Second, this was a single-center study with a small sample size, which may make our findings inconclusive. Third, the dose of each component used was subjective and empirical, and their mechanisms of action together as a cocktail were unclear, which may have affected the efficacy and safety of this cocktail treatment. In addition, the treatment procedure also prolonged the operation time. Finally, due to the retrospective nature of the control group, data on pre-stroke therapies were unavailable. Given the influence of pre-stroke therapies on safety outcomes, particularly anticoagulants, antiplatelets, antihypertensive medications, and statins, they should be considered in future studies.

## Conclusion

Intra-arterial administration of argatroban, dexamethasone, and edaravone following successful EVT reperfusion may be safe and feasible, but it did not improve the 90-day functional outcomes in anterior circulation AIS-LVO patients. However, the numerically higher probability of a favorable outcome and safety in the INSIST-CT group suggests that this cocktail therapy, targeting multiple pathophysiological processes after successful reperfusion, may be a promising strategy. A prospective multicenter randomized trial is warranted.

## Data Availability

The raw data supporting the conclusions of this article will be made available by the authors, without undue reservation.
