# Supplementary material for: Intra-arterial cocktail therapy for patients with anterior circulation large vessel occlusion who achieved endovascular reperfusion
Source: Front Neurol. 2024 Dec 6;15:1450156. doi: 10.3389/fneur.2024.1450156 (PMC11660178; doi:10.3389/fneur.2024.1450156)
Supplement: Supplementary file 1 [file Data_Sheet_1.docx]

**PROTOCOL**

Version 1.0 of October 06, 2021

**Improving Neuroprotective Strategy for Ischemic Stroke with sufficient recanalization after Thrombectomy by intra-arterial Cocktail Therapy (INSIST-CT): a prospective, single arm, pilot study**

**Principal Investigator:**

**Prof Hui-Sheng Chen**

Department of Neurology

General Hospital of Northern Theatre Command

83 Wenhua Rd

Shenyang, China

Tel: 86-024-28897511

Fax: 86-024-28897495

E-mail: chszh@aliyun.com

**Table of Contents**

[Abstract 4](#_Toc99443613)

[1.Background and current state of knowledge 8](#_Toc99443615)

[2.Objectives of the trial 9](#_Toc99443616)

[2.1 Hypothesis tested 9](#_Toc99443617)

[2.2 Primary objective 9](#_Toc99443618)

[2.3 Secondary objectives 9](#_Toc99443619)

[3. Design and selection of patients 10](#_Toc99443620)

[3.1 Trail plan 10](#_Toc99443621)

[3.2 Selection criteria: 12](#_Toc99443622)

[3.3 Duration of participation for each subject: 13](#_Toc99443623)

[3.4 Randomization and measures to reduce bias: 13](#_Toc99443624)

[4 Plan and conduct of the trial 13](#_Toc99443625)

[4.1 Clinical trial flow chart 13](#_Toc99443626)

[4.2 Study completion 16](#_Toc99443627)

[4.3 Study termination 16](#_Toc99443628)

[5. Treatment administered to included subjects 16](#_Toc99443632)

[5.1 Description of the treatment required to conduct the study 16](#_Toc99443633)

[5.2 Permitted and prohibited medical drugs and treatments in the study 16](#_Toc99443634)

[6. Outcome measurements 17](#_Toc99443635)

[6.1 Primary efficacy outcome 17](#_Toc99443636)

[6.2 Secondary efficacy outcomes 17](#_Toc99443637)

[6.3 Explotory outcomes 18](#_Toc99443638)

[6.4 Safety outcomes 18](#_Toc99443638)

[6.5 Study procedure 18](#_Toc99443639)

[6.6 Study periods 18](#_Toc99443640)

[7. Description of safety assessment parameters 20](#_Toc99443641)

[7.1 Evaluate clinical safety: 20](#_Toc99443642)

[7.2 Safety assessment 21](#_Toc99443643)

[7.3 Safety report 26](#_Toc99443644)

[7.4 Data Safety Monitoring Committee 26](#_Toc99443644)

[8. Statistics 28](#_Toc99443645)

[8.1 Sample size 28](#_Toc99443646)

[8.2 Statistical analysis plan 28](#_Toc99443647)

[8.3 Analysis population 29](#_Toc99443648)

[9. Data Management and Monitoring 29](#_Toc99443648)
[Reference 31](#_Toc99443653)
[Appendix 3](#_Toc99443649)2

[Appendix 1：National Institutes of Health Stroke Scale (NIHSS) 3](#_Toc99443650)4

[Appendix 2：Modified Rankin Score (mRS) 41](#_Toc99443651)

[Appendix 3：TOAST classification 42](#_Toc99443652)

Abstract

| **Title** | Improving Neuroprotective Strategy for Ischemic Stroke with sufficient recanalization after Thrombectomy by intra-arterial Cocktail Therapy (INSIST-CT): a prospective, single arm, pilot study |
| --- | --- |
| **Principle Centre** | General Hospital of Northern Theatre Command |
| **Sponsor** | Cerebrovascular Disease Collaboration Innovation Alliance (CDCIA) – Liaoning |
| **Objective** | To explore the safety, feasibility and efficacy of thrombectomy with sufficient recanalization bridged by intra-arterial cocktail therapy in acute ischemic stroke patients. |
| **Efficacy Outcome** | **Primary outcome：**   1. Proportion of modified Rankin Score (mRS, 0–2) at 90±7 days after randomization.   **Secondary outcomes：**   1. Proportion of mRS (0–1) at 90±7 days after randomization; 2. Occurrence of early neurological improvement within 48±12 hours. |
| **Safety Outcome** | Occurrence of symptomatic intracranial haemorrhage within 48±12 hours. |
| **Trial Design** | This is a prospective, single arm, open label, blinded endpoint, and single-centre trial. Follow-up will be performed at baseline, 48±12 hours, 10±2 days, and 90±7 days after randomization. All the investigators and outcome assessors will be masked to the allocation assignment and follow-up. |
| **Trial Population** | Acute anterior circulation large vessel occlusion patients with successful recanalization after endovascular treatment |
| **Sample Size** | 30 |
| **Inclusion criteria** | 1. Age ≥18 years. 2. Patients who presented with acute ischemic stroke and a large vessel occlusion in the anterior circulation and met the criteria of mechanical thrombectomy. 3. Sufficient recanalization (mTICI 2b-3) within 7 hours of stroke onset. 4. Signed informed consent. |
| **Exclusion criteria** | **Excluded**   1. insufficient recanalization (mTICI < 2a) after endovascular treatment; 2. Haemorrhagic stroke: cerebral haemorrhage, subarachnoid haemorrhage. 3. Coagulation disorders, systematic haemorrhagic tendency, thrombocytopenia （ <100000/mm3）. 4. Severe hepatic or renal dysfunction, increase in ALT or AST (more than 2 times of upper limit of normal value), increase in serum creatinine (more than 1.5 times of upper limit of normal value) or requiring dialysis. 5. Severe uncontrolled hypertension (systolic blood pressure over 200mmHg or diastolic blood pressure over 110 mmHg). 6. Patients with contraindication or allergic to any ingredient of drugs in our study. 7. Patients not suitable for the study considered by researcher. |
| **Trial Cycle** | All included patients will be followed up at 48 ± 12 hours, 10 ± 2 days, and 90 ± 7 days after enrolment. |
| **Treatment Regimens** | All enrolled patients will receive intra-arterial administration of argatroban (0.2-0.3 mg/min), dexamethasone (0.1 mg/min) and edaravone (0.3 mg/min) for 30 to 60 minutes after the end of thrombectomy. |
| **Procedure** | **Screening period:** On day 0 (baseline period), it is necessary to complete enrolment screening, and collect demographic characteristics, medical history (including history of hypertension, diabetes and drug treatment history), brain imaging (computer tomography, computed tomography angiography or magnetic resonance imaging), neurological measurements (NIHSS score, mRS score), haematological examination (blood routine, blood glucose, hepatic and renal function, coagulation routine, urine routine, electrocardiogram, etc) and other information.  **Treatment period:** Intra-arterial administration of argatroban (0.2-0.3 mg/min), dexamethasone (0.1 mg/min) and edaravone (0.3 mg/min) for 30 to 60 minutes after the end of thrombectomy.  **Follow-up period:** NIHSS score will be assessed at 48±12 hours, and 10 ± 2 days after enrolment. At 48±12 hours, patients will receive brain computer tomography or magnetic resonance imaging. The mRS score will be assessed at 90 ± 7 days. All concomitant medications, adverse events, stroke recurrence and other vascular events of each visit are recorded since the last visit.  All the adverse events of included subjects should be recorded and tracked until properly resolved.  All the serious adverse events of included subjects should be recorded and tracked, even if the subjects have finished the trial, until the events are resolved, or stabilization judged by the investigator. |
| **Planed Duration** | 24 months |

**1.Background and current state of knowledge**

Acute ischemic stroke (AIS) accounts for approximately 85% of all stroke events and is one of the leading causes of morbidity and mortality worldwide, especially in patients with large vessel occlusion (LVO) [1,2] Currently, rapid recanalization of occluded arterial vessels by endovascular treatment (EVT) has been the most effective treatments in this population [3]. A harsh truth was that only 46.0% patients achieved functional independence although there was about 80% successful recanalization of LVO [3]. The reasons for this phenomenon are generally attributed to the re-occlusion of the vessel, haemorrhagic transformation after EVT and no re-flow in the cerebral microcirculation [4]. Among these reasons, this no re-flow phenomenon may be a major contributor to the poor prognosis. Previous studies have shown that the mechanisms underlying no-reflow are multiple, including endothelial swelling, pericyte constriction, microthrombus formation, neuroinflammation, oxidative stress, and disruption of the blood-brain barrier [5-10].

Based on the above discussion, we argue that intra-arterial treatments of argatroban, edaravone, and glucocorticoids may safely improve clinical outcomes in AIS-LVO patients with successful recanalization who received EVT given their multiple actions. For example, argatroban could prevent and reduce microthrombus formation; edaravone would scavenge free radicals; and glucocorticoids could inhibit neuroinflammatory response and reduce endothelial oedema. In this context, this trial is designed to explore the safety, feasibility and possible efficacy of sufficient endovascular recanalization bridged by intra-arterial cocktail therapy in this population.

**2.Objectives of the trial**

**2.1 Hypothesis tested**

This study intends to demonstrate that intra-arterial cocktail therapy could safely treat anterior circulation AIS-LCO patients with sufficient endovascular recanalization.

**2.2 Primary objective**

To test the hypothesis that intra-arterial cocktail therapy can safely improve favourable functional outcome at 90 days in anterior circulation AIS-LCO patients with sufficient endovascular recanalization.

**2.3 Secondary objectives**

1. To determine the proportion of excellent functional outcome at 90 days.
2. To determine occurrence of early neurological improvement within 48 hours.

**2.4 Safety objectives**

1. To determine occurrence of symptomatic intracranial haemorrhage.

**3. Design and selection of patients**

**3.1 Trail plan**

This is a prospective, pilot, single-arm, open label, blinded endpoint, and single centre trial.

All enrolled patients will receive intra-arterial administration of argatroban (0.2-0.3 mg/min), dexamethasone (0.1 mg/min) and edaravone (0.3 mg/min) for 30 to 60 minutes after successful recanalization. In addition, all patients will receive standard medical care as per national stroke guidelines.

Follow-up will be performed at baseline, 48±12 hours, 10 ± 2 days, and 90 ± 7 days, respectively.

All clinical assessments including NIHSS and mRS will be evaluated by certified assessors according to a standardized procedure manual. The primary endpoint (mRS at 90 days) will be mainly evaluated, in person or by telephone interview (if in person interview is not possible), by one trained and certified staff.

**3.2 Selection criteria:**

**Inclusion criteria: in order to be eligible, the patients must meet all of the following criteria:**

1. Age ≥18 years.
2. Patients who presented with acute ischemic stroke and a large vessel occlusion in the anterior circulation and met the criteria of mechanical thrombectomy.
3. Sufficient recanalization (mTICI 2b-3) within 7 hours of stroke onset.
4. Signed informed consent.

**Exclusion criteria: in order to be included the patients must not have any of the following criteria:**

- - 1. Insufficient recanalization (mTICI < 2a) after endovascular treatment;
    2. Haemorrhagic stroke: cerebral haemorrhage, subarachnoid haemorrhage.
    3. Coagulation disorders, systematic haemorrhagic tendency, thrombocytopenia （ <100000/mm3）.
    4. Severe hepatic or renal dysfunction, increase in ALT or AST (more than 2 times of upper limit of normal value), increase in serum creatinine (more than 1.5 times of upper limit of normal value) or requiring dialysis.
    5. Severe uncontrolled hypertension (systolic blood pressure over 200mmHg or diastolic blood pressure over 110 mmHg).
    6. Patients with contraindication or allergic to any ingredient of drugs in our study.
    7. Patients not suitable for the study considered by researcher.

**Elimination criteria:**

1. Those who did not receive intra-arterial drugs after enrolment;

2. There is no laboratory examination record during the study;

3. Those who used other treatments that are prohibited and affect judgment of efficacy.

**Drop-out criteria:**

1. Subjects lost to follow-up and voluntarily requested to withdraw;

2. Due to any comorbidities, complications or special physiological changes, the subject is not suitable to continue the trial based on the investigator’s judgement;

3. Due to the disease deterioration, the subject is withdrawn from the trial based on the investigator’s judgement;

4. Poor compliance after enrolment.

**Suspension Criteria:**

Trial suspension means that the clinical trial has not finished as planned, and the trial is stopped in the middle period. The purpose of trial suspension is to protect the rights and interests of subjects, ensure the quality of the trial, and avoid unnecessary economic losses:

1. Proportion of serious adverse events during the trial is high than unexpected, and Data and Safety Monitoring Committee (DSMC) has the right to terminate the study unconditionally.

**3.3 Duration of participation for each subject:**

Each subject will be followed up in the trial for 3 months.

**3.4 Measures to reduce bias:**

Final follow-up will be done at 90 days, in person or by telephone, by the trained and certified assessors. Central adjudication of clinical and safety outcomes will be also done by assessors who are unaware of clinical details.

**4 Plan and conduct of the trial**

**4.1 Clinical trial flow chart**

| Period  Item | Screening | Treatment | Follow-up | | |
| --- | --- | --- | --- | --- | --- |
| Visit | 1 | 2 | 3 | 4 | 5 |
| Time | 0 day ^1^ | After the end of EVT | 48 ± 12 hours | 10 ± 2 days | 90 ± 7 days |
| Inclusion/Exclusion Criteria | x |  |  |  |  |
| Sign informed consent | x |  |  |  |  |
| Demographic characteristics^2^ | x |  |  |  |  |
| Medical history | x |  |  |  |  |
| Physical examination | x |  | x | x |  |
| Brain CT/MRI | x |  | x |  |  |
| TOAST classification |  |  |  | x |  |
| ECG（12 lead） | x |  |  |  |  |
| 24h ambulatory ECG ^3^ |  |  |  | x ^3^ |  |
| Blood routine ^4^ | x |  |  |  |  |
| Urine routine ^5^ | x |  |  |  |  |
| Blood biochemistry ^6^ | x |  |  |  |  |
| Coagulation routine ^7^ | x |  |  |  |  |
| NIHSS score | x |  | x | x |  |
| mRS score | x |  |  |  | x |
| Concomitant medication |  | x | x | x | x |
| Adverse events |  | x | x | x | x |
| Stroke recurrence and other vascular events ^8^ |  | x | x | x | x |

1. Day 0: limited to the period from the onset of stroke to the time before randomization;
2. Demographic characteristics: The age of the subjects is calculated based on the identification (ID) card information;
3. 24h ambulatory electrocardiogram: required to be completed within 10 days of admission;
4. Blood routine: including total number of red blood cells (RBC), total number of white blood cells (WBC), platelet count (PLT), haemoglobin (HGB). The results are measured within 24 hours before screening;
5. Urine routine: including urine red blood cells (NRBC), urine white blood cells (NWBC), urine protein (PRO), and urine sugar (GLU). The results are measured within 24 hours before screening;
6. Blood biochemistry: Hepatic function tests: alanine aminotransferase (ALT), aspartate aminotransferase (AST), total bilirubin (TBIL), direct bilirubin (DBIL); Renal function tests: serum urea nitrogen (BUN), Creatinine (Cr); Blood Glucose (GLU); Lipid, including total cholesterol (TC), triglycerides (TG), high density cholesterol (HDL), low density cholesterol (LDL). The results are measured within 24 hours before screening;
7. Coagulation examination: prothrombin time (PT), activated partial thromboplastin time (APTT), fibrinogen (FIB), thrombin time (TT). The results are measured within 24 hours before screening;
8. Stroke recurrence and other vascular events: occurrence of stroke recurrence and other vascular events within 90 days from randomization.

**4.2 Study completion**

The study is considered to be finished when the last visit of the last subject in the trial is completed.

**4.3 Study termination**

The sponsor holds the right to close the study centre or suspend the study at any time. The study centre should be closed after the study is completed. The centre is considered closed after all required documentation and study supplies have been collected and a centre closure visit has been conducted.

Investigators can also request to suspend the study, but they must give reasonable reasons in advance.

Reasons for the sponsor or investigator to close the centre in advance may include, but are not limited to:

1. Investigator fails to comply with the requirements of the study protocol, IEC/IRB or local regulatory authorities, sponsor's operating procedures or GCP guidelines;
2. Security considerations;
3. Investigators are not recruiting enough subjects.

**5. Treatment administered to included subjects**

**5.1 Description of the treatment required to conduct the study**

All enrolled patients will receive intra-arterial administration of argatroban (0.2-0.3 mg/min), dexamethasone (0.1 mg/min) and edaravone (0.3 mg/min) for 30 to 60 minutes after successful recanalization. In addition, all patients in the intervention arm will receive standard medical care as per national stroke guidelines.

**5.2 Permitted and prohibited medical drugs and treatments in the study**

There are no specific medical treatments for this study. The treatments are based on the current guidelines.

**6. Outcome measurements**

**6.1 Primary efficacy outcome**

Proportion of mRS (0–2) at 90±7 days after randomization.

**6.2 Secondary efficacy outcomes**

1. Proportion of mRS (0–1) at 90±7 days after randomization;
2. Occurrence of early neurological improvement (ENI) within 48±12 hours;

**6.3 Safety outcomes**

Occurrence of symptomatic intracranial haemorrhage within 48±12 hours.

Note:

- ENI is defined as more than 4-point decrease in NIHSS within 48±12 hours;
- Sympotmatic intracranial hemorrhage is defined as an increase in the NIHSS score of ≥4 points as a result of the intracranial hemorrhage according to the ECASS-3 study [11].

**6.5 Study procedure**

**
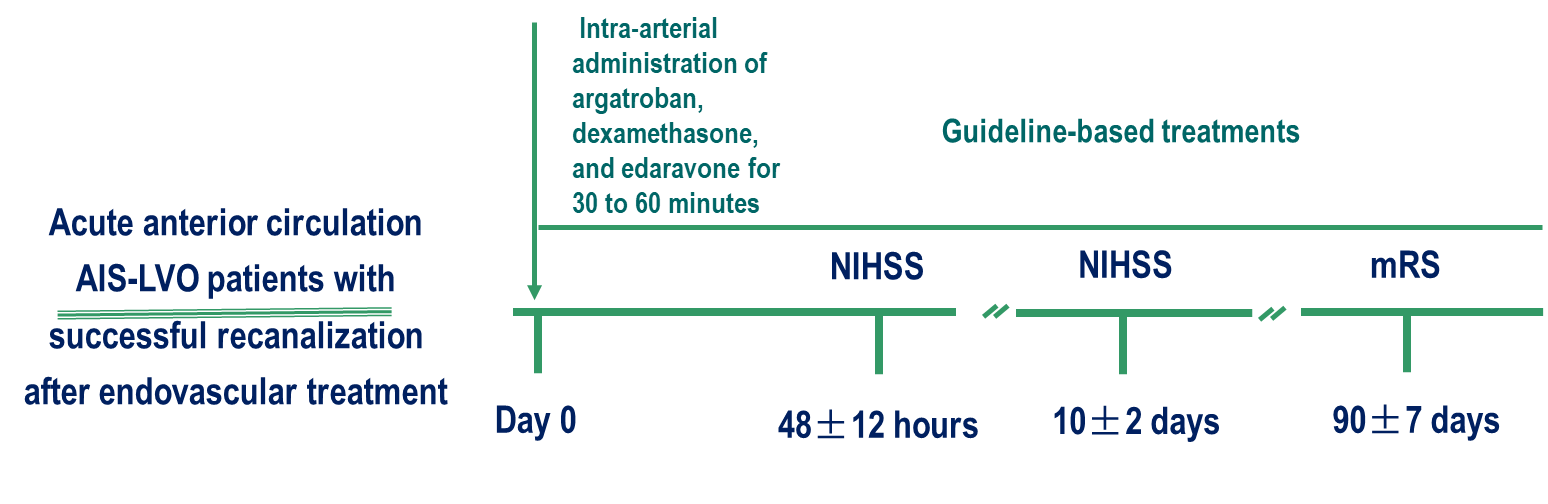
**

**6.6 Study periods**

**Screening period:** On day 0 (baseline period), it is necessary to complete enrolment screening, and collect demographic characteristics, medical history (including history of hypertension, diabetes and drug treatment history), brain imaging (computer tomography, computed tomography angiography or magnetic resonance imaging), neurological measurements (NIHSS score, mRS score), hematological examination (blood routine, blood glucose, hepatic and renal function, coagulation routine, urine routine, electrocardiogram, etc) and other information.

**Treatment period:** All enrolled patients receive intra-arterial drugs. The perioperative data including time and dose of intra-arterial drugs, LVO recanalization time, mTICI grade, etc. will be recorded and cranial CT will be performed immediately after the procedure.

**Follow-up period:** NIHSS score will be assessed at 48±12 hours, and 10 ± 2 days. Repeated brain CT will be performed at 48±12 hours. The mRS score will be assessed at 90 ± 7 days. All concomitant medications, adverse events, stroke recurrence and other vascular events of each visit will be recorded since the last visit.

All the adverse events of included subjects should be recorded and tracked until properly resolved.

All the serious adverse events of included subjects should be recorded and tracked, even if the subjects have finished the trial, until the events are resolved, or stabilization judged by the investigator.

**7. Description of safety assessment parameters**

**7.1 Evaluate clinical safety:**

Physical examinations, including neurological assessments, imaging studies, will be performed at baseline, 48 ± 12 hours, 10 ± 2 days, and 90 ± 7 days after enrolment; adverse event of each visit will be collected after baseline visit.

**Adverse events monitoring**

All information on adverse events, whether mentioned by subjects, discovered by investigators, or discovered through physical examination, laboratory tests, and other methods, should be recorded on the adverse events page of electronical case report form and handled in accordance with appropriate regulations and report.

**Adverse Event (AE）**

The adverse event (AE) is any adverse medical event that occurs in a study subject during the course of a study that is not necessarily related to this treatment. Thus, the adverse event (AE) can be any unfavourable and unexpected sign (including abnormal laboratory findings), symptom or disease temporally related to the use of study treatments, regardless of whether it is related to the study treatments or not.

It is included that any events that are new or that have worsened in severity and frequency from baseline, including abnormal results from diagnostic methods such as laboratory tests.

Note: Adverse event collection begins with signed informed consent.

**Serious Adverse Event (SAE）**

Serious adverse event (SAE) is any adverse medical event at any dose that meets one or more of the following criteria:

• cause death (note: death is a consequence, not an event);

• Life-threatening (Note: "Life-threatening" means that the subject is in immediate danger of death at the time of the event, not the assumption that death would have occurred if the event is more severe);

• Cause significant or permanent disability or impairment of organ function;

• Teratogenic and birth defects;

• Causing or prolonging hospitalization;

• Significant medical event or need for intervention (occur without treatment)’

Note: Any event requiring hospitalization (or prolonged hospitalization) that occurs during the course of the subject's participation in the study must be reported as a serious adverse event. The following circumstances leading to hospitalization are not considered serious adverse events:

• Hospitalization for social reasons other than adverse events

• Hospitalization for a scheduled surgery or other treatment or examination prior to study entry (must be recorded on the case report form)

• Hospitalization for elective surgery or treatment or examination due to anticipated disease progression

**7.2 Safety assessment**

For all adverse events in clinical studies, the following factors must be evaluated for safety:

• Severity criteria for SAE

• The causal relationship between the event and the investigational drugs

• Severity of incident

• Anticipation of events

**Causal relationship between events and the study treatments**

Regardless of serious adverse events or non-serious adverse events, the investigator must evaluate the relevance of the event to the use of the study drugs according to the following criteria:

**Evaluating association between adverse events and study treatments Criteria**

| **5-level classification** | **Judgment criteria** |
| --- | --- |
| Definitely related | The time of initiation of use is reasonably related to the time of occurrence of the adverse event; the adverse event corresponds to the known adverse reaction type of the study drugs; the occurrence of the adverse event cannot be explained by factors other than the study drugs; the adverse event reduce or disappear after study drug dose reduction or discontinuation; similar adverse events (non-essential) can occur with re-use of study drug. |
| Probably related | The time of initiation of use is reasonably related to the time of occurrence of the adverse event; the adverse event corresponds to the known adverse reaction type of the study drug; the occurrence of the adverse event can or cannot be explained by factors other than the study drug; the adverse event can or cannot reduce or disappear after study drug dose reduction or discontinuation; it is not sure whether similar adverse events (non-essential) can occur with re-use of study drug. |
| Probably unrelated | The time of initiation of use is not reasonably related to the time of occurrence of the adverse event; the adverse event corresponds to the known adverse reaction type of the study drug; the occurrence of the adverse event can or cannot be explained by factors other than the study drug; the adverse event can or cannot reduce or disappear after study drug dose reduction or discontinuation; it is not sure whether similar adverse events (non-essential) can occur with re-use of study drug. |
| Definitely unrelated | The time of initiation of use is not reasonably related to the time of occurrence of the adverse event; the adverse event corresponds to the known adverse reaction type of the study drug; the occurrence of the adverse event can or cannot be explained by factors other than the study drug; the adverse event can or cannot reduce or disappear after study drug dose reduction or discontinuation; similar adverse events (non-essential) cannot occur with re-use of study drug. |
| Unjudged | The judgment cannot be made due to incomplete, contradictory information or the fact that the information cannot be supplemented and verified. |

**Table of Correlation Evaluation Criteria for Adverse Events and Drug Use**

|  | Definitely related | Probably related | Probably unrelated | Definitely unrelated | Unjudged |
| --- | --- | --- | --- | --- | --- |
| Have a reasonable chronological order with the study drug | ＋ | ＋ | ＋ | － | ? |
| For the known reaction type of the study drug | ＋ | ＋ | － | － | ? |
| Explanation for reasons other than available research equipment | － | ± | ± | ＋ | ？ |
| Responses lessen after decompression of study drug | ＋ | ± | ± | － | ? |
| The reaction reappears after the study drug is reused | + or ? | ? | ? | － | ? |

Note: "+" in the table is affirmative; "-" is negative; "±" is difficult to affirm or deny; "?" indicates that the situation is unknown.

**Severity Criteria**

Severity should be assessed according to the following graded descriptions:

• Mild: No symptoms or discomfort; does not interfere with daily activities and function; usually does not require medication to relieve symptoms.

• Moderate: Symptoms cause significant discomfort; daily activities and function are affected; study participation can be continued; intervention required to relieve symptoms.

• Severe: Severe causes severe discomfort; symptoms result in loss of function and significantly interfere with daily activities; in severe cases, study drug can be discontinued; symptomatic treatment and/or hospitalization are required.

The investigator should use clinical judgment to assess the severity of the event (e.g., abnormal laboratory results) from the subject's direct experience.

**7.3 Safety report**

**All adverse events**

All adverse events, regardless of their severity, nature of severity, or their causal relationship to study drug, from the time of signed informed consent until the last follow-up visit (90 days after randomization), are to be recorded in medical terms on the clinical research form. When symptoms and signs are caused by common causes, a diagnosis of the disease should be given where possible (e.g., cough, runny nose, sneezing, sore throat, and headache should be reported as "upper respiratory tract infection"). The investigator must make a judgment on the causal relationship between the adverse event and the study drug and record it on the clinical research form. All drugs for adverse events should be documented in the original medical record and reported as requested by the sponsor.

All serious adverse events in clinical research must be reported to the ethics committee of the centre, the principal investigator of the centre, the sponsor, the contract research organization, the research team leader unit, and the adverse reaction monitoring centre within 24 hours; Report to the ethics committee and data and safety monitoring committee of the team leader unit within 7 natural days after being informed. Investigators must complete a serious adverse event form to record the time, severity, duration, actions taken and outcomes of serious adverse events. Any follow-up information for serious adverse events should also be reported in writing within 24 hours according to the above process.

All serious adverse events that do not resolve at study termination or subject early withdrawal must be followed up to any of the following:

- 1. Incident mitigation
  2. Events are stable
  3. If the baseline value is known, the event returns to the baseline value
  4. Events can be attributed to other treatments or factors not related to study
  5. It is unlikely that any further information will be available (subject or physician refuses to provide further information and remains lost to follow-up after various follow-up attempts)

**Death**

Death is the result of an event. The death of a subject in a clinical trial, regardless of whether the event is expected or treatment-related, would be considered a serious adverse event. Events leading to death should be recorded in medical terms and reported on the eCRF. All causes of death (death diagnoses) must be reported as serious adverse events. Investigators should make every effort to obtain and send death certificates and autopsy reports to designated personnel.

**Pregnancy**

If the researcher finds that the subject is pregnant during the research, the researcher should fill out the "Pregnancy Incident Report Form" within 24 hours after learning and report it to the clinical research supervisor, and notify the project leader by phone

Abnormal pregnancy results, considered serious adverse events, should be reported in accordance with the Serious Adverse Event Reporting Procedure.

**Abnormal laboratory test results**

During the course of the study, when the results of laboratory tests meet the following conditions, they must be regarded as adverse events and recorded in medical terms on the adverse events of the CRF:

1. Accompanying clinical symptoms
2. Causing a change in the trial protocol (eg treatment interruption or discontinuation)
3. Leading to a change in medical intervention or concomitant treatment
4. Clinically significant as judged by the investigator (medical and scientific methods should be used to judge whether an isolated laboratory abnormality is an adverse event)

When the adverse event meets the serious criteria, it should be reported in accordance with the serious adverse event reporting procedure.

**7.4 Data Safety Monitoring Committee (DSMC)**

An independent DSMC will be chaired by a neurologist, and include two neurologists and an independent statistician. DSMC will perform data verification every six months in this study. DSMC will recommend to terminate or continue the trial. The primary purpose of the termination is to protect the rights and interests of the patients and to avoid unnecessary economic losses.

**8. Statistics**

**8.1 Sample size**

Given that this was a pilot, single-arm study, no formal sample size calculation was performed. A total of 30 patients was set based on the recommendation of the Steering Committee.

**8.2 Statistical analysis plan**

Student’s t-test or Mann-Whitney U test will be used for comparison of continuous variables based on their normality, and Pearson 's χ² test will be performed for categorical variables. To compare the outcomes between two groups, logistic regression model will be used for the analyses of 90-day mRS, early neurological improvement, symptomatic intracranial hemorrhage and intracranial hemorrhage, generalized linear model for change in NIHSS score, and COX regression model for mortality at 90 days. To minimize bias in the group comparison, we will perform propensity score matching (PSM) and inverse probability of treatment weighting (IPTW) analysis accounting for multiple confounders. Propensity score (PS) will be calculated using a logistic regression model adjusted for age, baseline NIHSS, pre-treatment with IVT, MT passes and occlusion location, and performed 1:1 PSM based on the nearest-neighbor matching with a caliper width of 0.2 of the standard deviation of PS. IPTW will be estimated based on the PS to create a synthetic sample with baseline characteristics independent of treatment assignment, and further conducted IPTW-weighted analysis. All statistical analyses will be performed using SPSS software (version 25.0) and R software (version 4.3.2), and two-sided test with *P*<0.05 will be considered statistically significant.

**9. Data Management and Monitoring**

**9.1 Training of study site personnel**

Before the first patient is entered into the study, the sponsor (Cerebrovascular Disease Collaboration Innovation Alliance (CDCIA) – Liaoning) will organize and train all study personnel (medical, nursing and other staff), including the trial protocol, investigator’s brochure, the evaluation of related scales, treatment management, SAE report procedure, etc. For key endpoint “mRS”, a structured interview for the assessment will be used, and recorded by smartphone. The Principal Investigator will ensure that appropriate training relevant to the study cover all of these staff.

**9.2 Monitoring of the study**

During the study, staffs from CDCIA will have regular contacts with the study sites, including visits to:

- Provide the related information and support to the investigator(s)
- Confirm that the investigational team is adhering to the protocol and data are being accurately and timely recorded
- Confirm that the responsibility of study treatment management is being implemented
- Perform source data verification including deferred informed consent, laboratory results, neuroimaging data, clinical data, and neurological function evaluation (NIHSS, mRS) at baseline and follow-up

The staff will be available whenever the investigator or other personnel at the center needs information and advice about the study.

**Reference**

1 Hankey, G. J. Stroke. Lancet 389, 641-654, doi:10.1016/S0140-6736(16)30962-X (2017).

2 Langhorne, P., Bernhardt, J. & Kwakkel, G. Stroke rehabilitation. Lancet 377, 1693-1702, doi:10.1016/S0140-6736(11)60325-5 (2011).

3 Goyal, M. et al. Endovascular thrombectomy after large-vessel ischaemic stroke: a meta-analysis of individual patient data from five randomised trials. Lancet 387, 1723-1731, doi:10.1016/S0140-6736(16)00163-X (2016).

4 Rezkalla, S. H. & Kloner, R. A. No-reflow phenomenon. Circulation 105, 656-662, doi:10.1161/hc0502.102867 (2002).

5 Yemisci, M. et al. Pericyte contraction induced by oxidative-nitrative stress impairs capillary reflow despite successful opening of an occluded cerebral artery. Nature medicine 15, 1031-1037, doi:10.1038/nm.2022 (2009).

6 Kloner, R. A., King, K. S. & Harrington, M. G. No-reflow phenomenon in the heart and brain. American journal of physiology. Heart and circulatory physiology 315, H550-H562, doi:10.1152/ajpheart.00183.2018 (2018).

7 Mohamed Mokhtarudin, M. J. & Payne, S. J. Mathematical model of the effect of ischemia-reperfusion on brain capillary collapse and tissue swelling. Mathematical biosciences 263, 111-120, doi:10.1016/j.mbs.2015.02.011 (2015).

8 Kutuzov, N., Flyvbjerg, H. & Lauritzen, M. Contributions of the glycocalyx, endothelium, and extravascular compartment to the blood-brain barrier. Proceedings of the National Academy of Sciences of the United States of America 115, E9429-E9438, doi:10.1073/pnas.1802155115 (2018).

9 Myers, G. J. & Wegner, J. Endothelial Glycocalyx and Cardiopulmonary Bypass. The journal of extra-corporeal technology 49, 174-181 (2017).

10 VanTeeffelen, J. W., Brands, J. & Vink, H. Agonist-induced impairment of glycocalyx exclusion properties: contribution to coronary effects of adenosine. Cardiovascular research 87, 311-319, doi:10.1093/cvr/cvq114 (2010).

11 Heard, B. J. et al. Single intra-articular dexamethasone injection immediately post-surgery in a rabbit model mitigates early inflammatory responses and post-traumatic osteoarthritis-like alterations. Journal of orthopaedic research : official publication of the Orthopaedic Research Society 33, 1826-1834, doi:10.1002/jor.22972 (2015).

12. Hacke W, et al. Thrombolysis with alteplase 3 to 4.5 hours after acute ischemic stroke. N Engl J Med. 2008;359:1317–1329.

**Appendix**

**Appendix 1：National Institutes of Health Stroke Scale (NIHSS)**

|  | Check | Score | Point |
| --- | --- | --- | --- |
| 1a | Level of Consciousness: The investigator must choose a response if a full evaluation is prevented by such obstacles as an endotracheal tube, language barrier, orotracheal trauma/bandages. A 3 is scored only if the patient makes no movement (other than reflexive posturing) in response to noxious stimulation. | 0 = Alert; keenly responsive.  1 = Not alert; but arousable by minor stimulation to obey, answer, or respond.  2 = Not alert; requires repeated stimulation to attend, or is obtunded and requires strong or painful stimulation to make movements (not stereotyped).  3 = Responds only with reflex motor or autonomic effects or totally unresponsive, flaccid, and areflexic. | — |
| 1b | LOC Questions: The patient is asked the month and his/her age. The answer must be correct - there is no partial credit for being close. Aphasic and stuporous patients who do not comprehend the questions will score 2. Patients unable to speak because of endotracheal intubation, orotracheal trauma, severe dysarthria from any cause, language barrier, or any other problem not secondary to aphasia are given a 1. It is important that only the initial answer be graded and that the examiner not "help" the patient with verbal or non-verbal cues. | 0 = Answers both questions correctly.  1 = Answers one question correctly.  2 = Answers neither question correctly. | — |
| 1c | LOC Commands: The patient is asked to open and close the eyes and then to grip and release the non-paretic hand. Substitute another one step command if the hands cannot be used. Credit is given if an unequivocal attempt is made but not completed due to weakness. If the patient does not respond to command, the task should be demonstrated to him or her (pantomime), and the result scored (i.e., follows none, one or two commands). Patients with trauma, amputation, or other physical impediments should be given suitable one-step commands. Only the first attempt is scored. | 0 = Performs both tasks correctly.  1 = Performs one task correctly.  2 = Performs neither task correctly. | — |
| 2 | Best Gaze: Only horizontal eye movements will be tested. Voluntary or reflexive (oculocephalic) eye movements will be scored, but caloric testing is not done. If the patient has a conjugate deviation of the eyes that can be overcome by voluntary or reflexive activity, the score will be 1. If a patient has an isolated peripheral nerve paresis (CN III, IV or VI), score a 1. Gaze is testable in all aphasic patients. Patients with ocular trauma, bandages, pre-existing blindness, or other disorder of visual acuity or fields should be tested with reflexive movements, and a choice made by the investigator. Establishing eye contact and then moving about the patient from side to side will occasionally clarify the presence of a partial gaze palsy. | 0 = Normal.  1 = Partial gaze palsy; gaze is abnormal in one or both eyes, but forced deviation or total gaze paresis is not present.  2 = Forced deviation, or total gaze paresis not overcome by the oculocephalic maneuver. | — |
| 3 | Visual: Visual fields (upper and lower quadrants) are tested by confrontation, using finger counting or visual threat, as appropriate. Patients may be encouraged, but if they look at the side of the moving fingers appropriately, this can be scored as normal. If there is unilateral blindness or enucleation, visual fields in the remaining eye are scored. Score 1 only if a clear-cut asymmetry, including quadrantanopia, is found. If patient is blind from any cause, score 3. Double simultaneous stimulation is performed at this point. If there is extinction, patient receives a 1, and the results are used to respond to item 11. | 0 = No visual loss.  1 = Partial hemianopia.  2 = Complete hemianopia.  3 = Bilateral hemianopia (blind including cortical blindness). | — |
| 4 | Facial Palsy: Ask – or use pantomime to encourage – the patient to show teeth or raise eyebrows and close eyes. Score symmetry of grimace in response to noxious stimuli in the poorly responsive or non-comprehending patient. If facial trauma/bandages, orotracheal tube, tape or other physical barriers obscure the face, these should be removed to the extent possible. | 0 = Normal symmetrical movements.  1 = Minor paralysis (flattened nasolabial fold, asymmetry on smiling).  2 = Partial paralysis (total or near-total paralysis of lower face).  3 = Complete paralysis of one or both sides (absence of facial movement in the upper and lower face). | — |
| 5 | Motor Arm: The limb is placed in the appropriate position: extend the arms (palms down) 90 degrees (if sitting) or 45 degrees (if supine). Drift is scored if the arm falls before 10 seconds. The aphasic patient is encouraged using urgency in the voice and pantomime, but not noxious stimulation. Each limb is tested in turn, beginning with the non-paretic arm. Only in the case of amputation or joint fusion at the shoulder, the examiner should record the score as untestable (UN), and clearly write the explanation for this choice. | 0 = No drift; limb holds 90 (or 45) degrees for full 10 seconds.  1 = Drift; limb holds 90 (or 45) degrees, but drifts down before full 10 seconds; does not hit bed or other support.  2 = Some effort against gravity; limb cannot get to or maintain (if cued) 90 (or 45) degrees, drifts down to bed, but has some effort against gravity.  3 = No effort against gravity; limb falls.  4 = No movement.  UN = Amputation or joint fusion, explain: _____________________  5a. Left Arm  5b. Right Arm | ____  ____ |
| 6 | Motor Leg: The limb is placed in the appropriate position: hold the leg at 30 degrees (always tested supine). Drift is scored if the leg falls before 5 seconds. The aphasic patient is encouraged using urgency in the voice and pantomime, but not noxious stimulation. Each limb is tested in turn, beginning with the non-paretic leg. Only in the case of amputation or joint fusion at the hip, the examiner should record the score as untestable (UN), and clearly write the explanation for this choice. | 0 = No drift; leg holds 30-degree position for full 5 seconds.  1 = Drift; leg falls by the end of the 5-second period but does not hit bed.  2 = Some effort against gravity; leg falls to bed by 5 seconds, but has some effort against gravity.  3 = No effort against gravity; leg falls to bed immediately.  4 = No movement.  UN = Amputation or joint fusion, explain: ________________  6a. Left Leg  6b. Right Leg | _____  _____ |
| 7 | Limb Ataxia: This item is aimed at finding evidence of a unilateral cerebellar lesion. Test with eyes open. In case of visual defect, ensure testing is done in intact visual field. The finger-nose-finger and heel-shin tests are performed on both sides, and ataxia is scored only if present out of proportion to weakness. Ataxia is absent in the patient who cannot understand or is paralyzed. Only in the case of amputation or joint fusion, the examiner should record the score as untestable (UN), and clearly write the explanation for this choice. In case of blindness, test by having the patient touch nose from extended arm position. | 0 = Absent.  1 = Present in one limb.  2 = Present in two limbs.  UN = Amputation or joint fusion, explain: ________________ | — |
| 8 | Sensory: Sensation or grimace to pinprick when tested, or withdrawal from noxious stimulus in the obtunded or aphasic patient. Only sensory loss attributed to stroke is scored as abnormal and the examiner should test as many body areas (arms [not hands], legs, trunk, face) as needed to accurately check for hemisensory loss. A score of 2, “severe or total sensory loss,” should only be given when a severe or total loss of sensation can be clearly demonstrated. Stuporous and aphasic patients will, therefore, probably score 1 or 0. The patient with brainstem stroke who has bilateral loss of sensation is scored 2. If the patient does not respond and is quadriplegic, score 2. Patients in a coma (item 1a=3) are automatically given a 2 on this item. | 0 = Normal; no sensory loss.  1 = Mild-to-moderate sensory loss; patient feels pinprick is less sharp or is dull on the affected side; or there is a loss of superficial pain with pinprick, but patient is aware of being touched.  2 = Severe to total sensory loss; patient is not aware of being touched in the face, arm, and leg. | — |
| 9 | Best Language: A great deal of information about comprehension will be obtained during the preceding sections of the examination. For this scale item, the patient is asked to describe what is happening in the attached picture, to name the items on the attached naming sheet and to read from the attached list of sentences. Comprehension is judged from responses here, as well as to all of the commands in the preceding general neurological exam. If visual loss interferes with the tests, ask the patient to identify objects placed in the hand, repeat, and produce speech. The intubated patient should be asked to write. The patient in a coma (item 1a=3) will automatically score 3 on this item. The examiner must choose a score for the patient with stupor or limited cooperation, but a score of 3 should be used only if the patient is mute and follows no one-step commands. | 0 = No aphasia; normal.  1 = Mild-to-moderate aphasia; some obvious loss of fluency or facility of comprehension, without significant limitation on ideas expressed or form of expression. Reduction of speech and/or comprehension, however, makes conversation about provided materials difficult or impossible. For example, in conversation about provided materials, examiner can identify picture or naming card content from patient’s response.  2 = Severe aphasia; all communication is through fragmentary expression; great need for inference, questioning, and guessing by the listener. Range of information that can be exchanged is limited; listener carries burden of communication. Examiner cannot identify materials provided from patient response.  3 = Mute, global aphasia; no usable speech or auditory comprehension. | — |
| 10 | Dysarthria: If patient is thought to be normal, an adequate sample of speech must be obtained by asking patient to read or repeat words from the attached list. If the patient has severe aphasia, the clarity of articulation of spontaneous speech can be rated. Only if the patient is intubated or has other physical barriers to producing speech, the examiner should record the score as untestable (UN), and clearly write an explanation for this choice. Do not tell the patient why he or she is being tested. | 0 = Normal.  1 = Mild-to-moderate dysarthria; patient slurs at least some words and, at worst, can be understood with some difficulty.  2 = Severe dysarthria; patient's speech is so slurred as to be unintelligible in the absence of or out of proportion to any dysphasia, or is mute/anarthric.  UN = Intubated or other physical barrier, explain:_________________________ | — |
| 11 | Extinction and Inattention (formerly Neglect): Sufficient information to identify neglect may be obtained during the prior testing. If the patient has a severe visual loss preventing visual double simultaneous stimulation, and the cutaneous stimuli are normal, the score is normal. If the patient has aphasia but does appear to attend to both sides, the score is normal. The presence of visual spatial neglect or anosagnosia may also be taken as evidence of abnormality. Since the abnormality is scored only if present, the item is never untestable. | 0 = No abnormality.  1 = Visual, tactile, auditory, spatial, or personal inattention or extinction to bilateral simultaneous stimulation in one of the sensory modalities.  2 = Profound hemi-inattention or extinction to more than one modality; does not recognize own hand or orients to only one side of space. | — |

**Appendix 2：****Modified Rankin Score (mRS)**

| Grade | Description |
| --- | --- |
| 0 | No symptoms |
| 1 | Symptoms without any incapacity (able to perform all usual activities) |
| 2 | Mild incapacity (unable to perform all usual activities but able to look after his/her affairs alone) |
| 3 | Moderate incapacity (requires assistance but walks alone) |
| 4 | Severe incapacity (requires assistance for walking and physical body needs) |
| 5 | Severe incapacity (bedbound, incontinent, permanent surveillance required) |
| 6 | Death |

**Appendix 3：****TOAST classification**

- - 1. Large atherosclerotic type: requires vascular imaging examination to confirm that the intracranial or extracranial large artery stenosis corresponding to the neurological deficit of cerebral infarction is more than 50% or occluded, and the vascular lesions are consistent with atherosclerotic changes; or there is intracranial or Indirect evidence of stenosis or occlusion of extracranial large arteries, such as imaging (CT or MRI) showing cerebral cortex, brainstem, cerebellum or subcortical infarction with a diameter of >1.5cm, and clinical manifestations are mainly signs of cortical damage, such as aphasia , changes in consciousness, body image disturbance, etc., or signs of brainstem and cerebellum damage. Evidence of at least one atherosclerotic stroke risk factor (such as advanced age, hypertension, hyperlipidaemia, etc.) or systemic atherosclerosis (such as plaque, coronary heart disease, etc.) is required. At the same time, cerebral infarction caused by cardio embolism should also be excluded, such as no acute infarction outside the stenosis > 50% or occlusion of intracranial or extracranial large arteries, and no high or moderate risk factors for cardioembolic stroke (see Brain Embolization chapter).
    2. Cardiogenic cerebral embolism type: the clinical manifestations and imaging studies are the same as those of large atherosclerotic type. This classification is supported if there is more than one vessel innervation or multisystem embolism. The presence of at least one high or moderate risk factor for cardioembolic stroke is required.
    3. Small artery occlusion type: There may be no obvious clinical manifestations or various lacunar syndromes, but no cerebral cortex involvement. The head CT or MRI is required to be normal, or the infarct diameter is less than 1.5cm.
    4. Other etiological types: refer to other rare aetiologies other than the above three types of clear etiology. Such as blood coagulation disorders, changes in blood components, vasculitis due to various reasons, vascular malformations, connective tissue diseases, dissecting aneurysms, fibrous dystrophy and cerebral infarction.
    5. Unexplained type: including two or more aetiologies, negative auxiliary examinations, no cause found, and insufficient auxiliary examinations.
