# Supplementary material for: Intra-arterial cocktail therapy for patients with anterior circulation large vessel occlusion who achieved endovascular reperfusion
Source: Front Neurol. 2024 Dec 6;15:1450156. doi: 10.3389/fneur.2024.1450156 (PMC11660178; doi:10.3389/fneur.2024.1450156)
Supplement: Supplementary file 2 [file Table_1.DOCX]

# Supplementary Materials

Table S1. Baseline characteristics after propensity score matching

| Variables | INSIST-CT group  (n=30) | Control group  (n=30) | *P* value | SMD |
| --- | --- | --- | --- | --- |
| Age, year, median (IQR) | 69 (58-76) | 71 (62-77) | 0.965 | 0.045 |
| Men, n (%) | 17 (56.7) | 25 (83.3) | 0.024 | 0.608 |
| Medical history, n (%) |  |  |  |  |
| Hypertension | 20 (66.7) | 20 (66.7) | 1.000 | <0.001 |
| Diabetes mellitus | 12 (40.0) | 7 (23.3) | 0.165 | 0.364 |
| Hyperlipidemia | 5 (16.7) | 6 (21.4) | 0.644 | 0.121 |
| Coronary heart disease | 8 (26.7) | 8 (26.7) | 1.000 | <0.001 |
| Atrial fibrillation | 19 (63.3) | 14 (46.6) | 0.194 | 0.340 |
| Prior stroke | 7 (23.3) | 9 (30.0) | 0.559 | 0.151 |
| Smoking, n (%) | 13 (43.3) | 16 (53.3) | 0.438 | 0.201 |
| Drinking, n (%) | 13 (43.3) | 15 (50.0) | 0.605 | 0.134 |
| Pre-stroke mRS, n (%) |  |  | 0.083 | 0.474 |
| 0 | 25 (83.3) | 29 (96.7) |  |  |
| 1 | 3 (10.0) | 1 (3.3) |  |  |
| 2 | 2 (6.7) | 0 (0) |  |  |
| NIHSS, median (IQR) | 16 (12-19) | 17 (13-19) | 0.302 | 0.339 |
| ASPECTS, median (IQR)† | 9 (8-10) | 9 (8-9) | 0.105 | 0.464 |
| Pre-treatment with IVT, n (%) | 4 (13.3) | 6 (20.0) | 0.488 | 0.180 |
| Duration, min, median (IQR) |  |  |  |  |
| OPT | 287 (187-470) | 407 (294-484) | 0.122 | 0.419 |
| GRT | 50 (43-58) | 50 (38-67) | 0.847 | 0.085 |
| ORT | 339 (241-524) | 455 (323-557) | 0.110 | 0.429 |
| EVT, n (%) |  |  |  |  |
| Stent retriever | 30 (100.0) | 30 (100.0) | 1.000 | <0.001 |
| Aspiration | 3 (10.0) | 1 (3.3) | 0.612 | 0.270 |
| Balloon angioplasty | 6 (20.0) | 5 (16.7) | 0.739 | 0.086 |
| Stenting angioplasty | 1 (3.3) | 3 (10.0) | 0.612 | 0.270 |
| MT passes, n, median (IQR) | 1 (1-2) | 1 (1-2) | 0.743 | 0.094 |
| Cause of vessel occlusion, n (%) | |  | 0.530 | 0.294 |
| Atherosclerosis | 7 (23.3) | 11 (36.7) |  |  |
| Cardioembolism | 17 (56.7) | 14 (46.7) |  |  |
| Other or unknown etiology | 6 (20.0) | 5 (16.7) |  |  |
| Occlusion location, n (%) |  |  | 0.530 | 0.294 |
| MCA | 22 (73.3) | 21 (70.0) |  |  |
| ICA | 8 (26.7) | 9 (30) |  |  |

Abbreviations: SMD, standardized mean difference; NIHSS, National Institute of Health Stroke Scale; ASPECTS, Alberta Stroke Program Early CT Score; IVT, intravenous thrombolysis; OPT, onset to puncture time; PRT, puncture to recanalization time; ORT, onset to recanalization time; EVT, endovascular treatment; MT, mechanical thrombectomy; MCA, middle cerebral artery; ICA, internal carotid artery.

†One missing data.

Matched for age, pre-treatment with IVT, baseline NIHSS, MT passes, Occlusion location with caliper width 0.2

Table S2. Baseline characteristics after inverse probability of treatment weighting

| Variables | INSIST-CT group (n= 285) | Control group (n= 291) | SMD |
| --- | --- | --- | --- |
| Age, year, median (IQR) | 64 (56-74) | 64 (56-72) | 0.080 |
| Men, n (%) | 175 (61.2) | 228 (78.4) | 0.381 |
| Medical history, n (%) |  |  |  |
| Hypertension | 195 (68.3) | 152 (52.2) | 0.332 |
| Diabetes mellitus | 115 (40.3) | 74 (25.6) | 0.317 |
| Hyperlipidemia | 46 (15.9) | 65 (22.9) | 0.175 |
| Coronary heart disease | 78 (27.2) | 54 (18.6) | 0.205 |
| Atrial fibrillation | 184 (64.6) | 122 (41.8) | 0.468 |
| Prior stroke | 43 (15.1) | 64 (21.8) | 0.173 |
| Smoking, n (%) | 141 (49.6) | 163 (56.0) | 0.129 |
| Drinking, n (%) | 152 (53.1) | 154 (53.0) | 0.003 |
| Pre-stroke mRS, n (%) | |  | 0.096 |
| 0 | 256 (89.7) | 269 (92.5) |  |
| 1 | 19 (6.8) | 15 (5.1) |  |
| 2 | 10 (3.5) | 7 (2.4) |  |
| NIHSS, median (IQR) | 14 (12-17) | 15 (12-17) | 0.027 |
| ASPECTS, median (IQR)‡ | 9 (8-10) | 9 (8-10) | 0.223 |
| Pre-treatment with IVT, n (%) | 72 (25.1) | 84 (28.9) | 0.086 |
| Duration, min, median (IQR) | |  |  |
| OPT | 310 (183-454) | 360 (240-500) | 0.237 |
| GRT | 50 (43-58) | 51 (39-80) | 0.463 |
| ORT | 359 (242-522) | 429 (310-567) | 0.302 |
| EVT, n (%) |  |  |  |
| Stent retriever | 285 (100.0) | 286 (98.2) | 0.194 |
| Aspiration | 27 (9.6) | 30 (10.2) | 0.020 |
| Balloon angioplasty | 49 (17.0) | 73 (25.1) | 0.200 |
| Stenting angioplasty | 10 (3.5) | 22 (7.5) | 0.177 |
| MT passes, n, median (IQR) | 2 (1-2) | 1 (1-2) | 0.011 |
| Cause of vessel occlusion, n (%) | |  | 0.451 |
| Atherosclerosis | 62 (21.7) | 118 (40.7) |  |
| Cardioembolism | 173 (60.5) | 118 (40.7) |  |
| Other or unknown etiology | 51 (17.9) | 54 (18.6) |  |
| Occlusion location, n (%) |  |  | 0.139 |
| MCA | 227 (79.5) | 214 (73.6) |  |
| ICA | 58 (20.5) | 77 (26.4) |  |

Abbreviations: SMD, standardized mean difference; NIHSS, National Institute of Health Stroke Scale; ASPECTS, Alberta Stroke Program Early CT Score; IVT, intravenous thrombolysis; OPT, onset to puncture time; PRT, puncture to recanalization time; ORT, onset to recanalization time; EVT, endovascular treatment; MT, mechanical thrombectomy; MCA, middle cerebral artery; ICA, internal carotid artery.

A standardized mean difference smaller than 0.1 was considered insignificant difference.

Weighted for age, pre-treatment with IVT, baseline NIHSS, MT passes, Occlusion location.
